# Microvascular Abnormalities, Inner Retina Thinning and Sectorial Optic Atrophy after Low-dose Stereotactic Radiotherapy for Neovascular Age-Related Macular Degeneration: A Case Report

**DOI:** 10.1055/a-2542-4073

**Published:** 2025-04-16

**Authors:** Angelica Rizzato, Helena Giannakaki-Zimmermann, Florian Weisskopf, Katja Hatz-Wurziger

**Affiliations:** 1 Vista Klinik AG, Binningen, Switzerland; 2 Eye Clinic, University Hospital Basel, Basel, Switzerland; 3 Medical Faculty, University Hospital Basel, Basel, Switzerland

## Introduction

Radiation therapy was under extensive investigation between 1996 and 2007 as a potential treatment for neovascular age-related macular degeneration (nAMD), owing to its anti-angiogenetic and antifibrotic effects 1 . Targeting the proliferating endothelial cells, the therapy aimed to slow the progression of macular neovessels associated with AMD 2 , 3 , 4 . However, the clinical studies on external beam radiation in nAMD have yielded inconsistent results, leading to concerns about its effectiveness and safety 5 .

Years later, the development of technologies capable of providing a more targeted delivery of radiation has renewed interest in this treatment approach as an adjuvant therapy in combination with intravitreal anti-vascular endothelial growth factor (anti-VEGF) injections aiming to reduce the frequency of injections. IRay stereotactic radiotherapy (Oraya Therapeutics Inc., Newark, CA, USA) delivered a low-energy X-ray radiation over a 4-mm retinal treatment zone in a single session, thus offering more precise and safer delivery methods. The INTREPID study, a randomized, double-masked, sham-controlled study, showed a significant reduction in the number of required intravitreal anti-VEGF injections over 2 years 3 . However, one of the main concerns with applying radiation to the eye is the potential development of radiation retinopathy. For stereotactic radiotherapy (SRT), 1- and 2-year follow-up data indicated low incidences of radiation retinopathy, including retinal microvascular abnormalities (MVAs) 3 , 4 , 5 . However, the incidence of MVAs increased to 30% after 3 years 6 . Unfortunately, the study took place in 2011, therefore it did not include optical coherence tomography angiography (OCT-A) in the investigation plan. There is a notable absence of long-term OCT-A follow-up for patients that received radiation treatment for nAMD in the current and past literature.

Although the potential for radiation retinopathy associated with radiotherapy is well established in the medical literature, the specific adverse effects of low-dose SRT on the optic nerve and inner retinal layers are less defined. To this point, to the best of our knowledge, there have been no instances reported of optic neuropathy linked to low-dose SRT in patients treated for nAMD 2 .

## Case Presentation

**Fig. 1:**
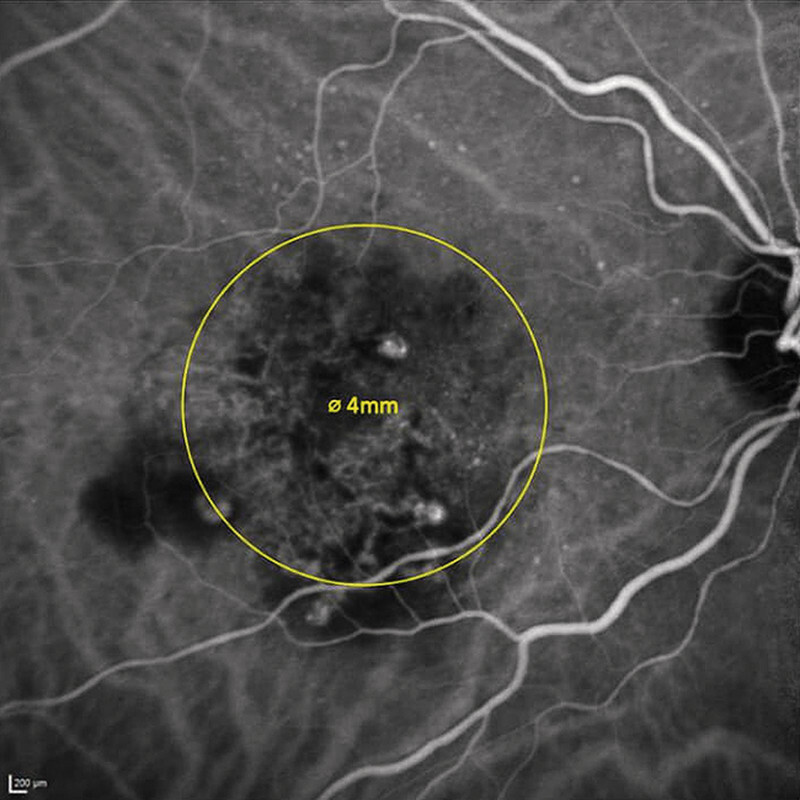
Application area of the total radiation dose of 16 Gy over a 4-mm area centered on the fovea.

**Fig. 2:**
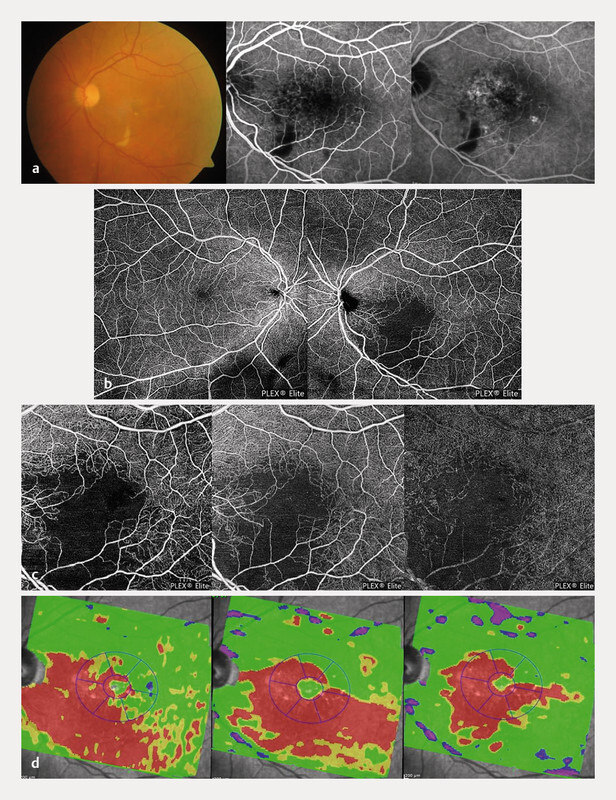
**a** Fundus photograph of the left eye showing the presence of cotton wool spots along the inferior temporal vascular arcade. On the right, early- and late-phase fluorescein angiographic images are depicted. Notice the blockage of the underlying hyperfluorescence due to the presence **of** cotton wool spots and a mild surrounding capillary leakage. **b** En face optical coherence tomography angiography (swept-source OCT-A Plex Elite 9000; Carl Zeiss Meditec, Oberkochen, Germany) 12 × 12-mm images showing both the superficial and the deep capillary plexus of both eyes. The left eye exhibits a wide area of non-perfusion with extension toward the foveal zone. **c** En face OCT-A 6 × 6-mm images of the left eye left to right: retinal vascular plexuses, superficial capillary plexus, and deep capillary plexus. The image highlights vascular defects in both the superficial and deep capillary plexus. **d** Deviation thickness maps of the posterior pole obtained with SD-OCT, from left to right: retinal nerve fiber layer thickness deviation map, ganglion cell layer thickness deviation map, inner nuclear layer thickness deviation map. A thinning of all three layers was observed in the perifoveal nasal and inferior macular regions.

A 68-year-old male was referred to our clinic with suspicion of active nAMD in the left eye. Comprehensive baseline diagnostics included visual acuity testing, intraocular pressure measurement, biomicroscopic fundus examination, spectral-domain OCT (SD-OCT; Spectralis, Heidelberg Engineering, Heidelberg, Germany), including macular volume scan, macular star and a 6-mm horizontal scan centered to the fovea, fluorescein angiography (FA), and indocyanine green angiography (ICG) with a 30° setting centered to the fovea (HRA2, Heidelberg Engineering, Heidelberg, Germany). Corrected distance visual acuity (VA) was 1.0 in the right eye and 0.3 in the left eye. nAMD with active MNV type 1 was diagnosed in the left eye and an intravitreal treatment with ranibizumab (Lucentis, Novartis, Basel, Switzerland) was initiated. The investigations did not reveal any abnormalities of the optic disc. First, the treatment followed a pro re nata (PRN) regimen, which was then adjusted to a treat-and-extend protocol. However, the patient showed an incomplete response to anti-VEGF treatment, with persistent subretinal fluid after 21 intravitreal injections and despite the 4-weekly interval. The patient was offered an adjuvant low-energy SRT with an IRAY system (Oraya Therapeutics Inc., Newark, CA, USA), whose mechanism of action 7 , along with the specific procedural approach employed, has been detailed above and in previous publications 8 . The entire radiating dose, amounting to a total of 16 Gy, was delivered over a 4-mm diameter area centered on the fovea ( Fig. 1 ) in a single session and with a radiation exposure time between 4 and 5 minutes. The treatment was conducted. After 6 months, a complete resolution of subretinal fluid was achieved (shown in Fig. 2 ). Subsequent recurrences of subretinal fluid accumulation led to a 4-weekly anti-VEGF treatment according to a treat-and-extend protocol, and a switch to aflibercept (Eylea, Bayer, Leverkusen, Germany) was then attempted.

**Fig. 3:**
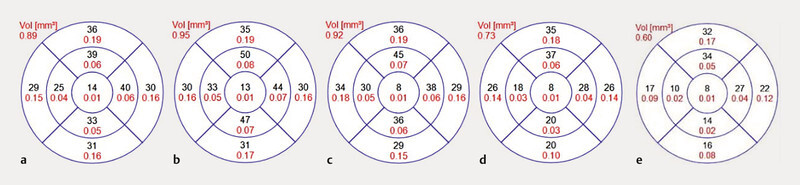
Macular ganglion cell layer thickness analysis (EDTRS grid) following a timeline. A developing atrophy over the years can be observed.

**Fig. 4:**
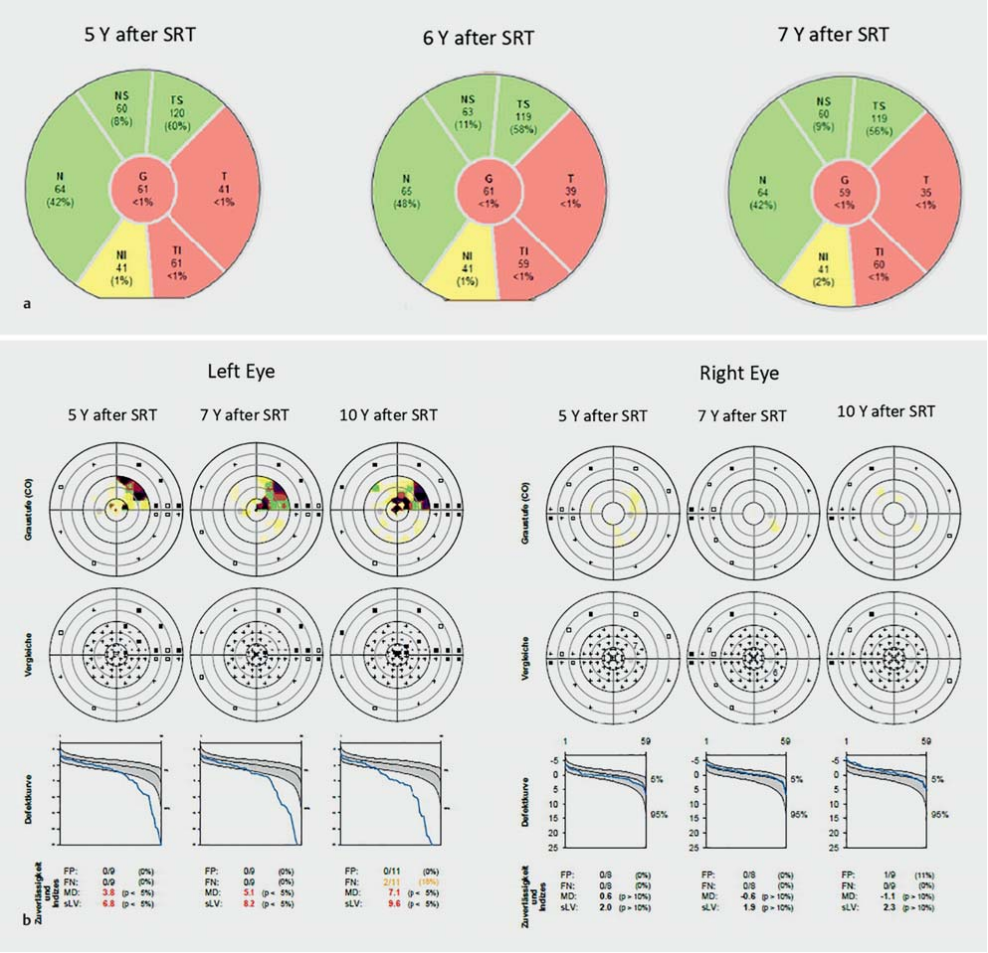
**a** Series of a peripapillary retinal nerve fiber layer (RNFL) optical coherence tomography scan of the left eye showing a thinning of the RNFL in the temporal quadrant. **b** Perimetric exams during follow-up at 5, 7, and 10 years after stereotactic radiotherapy. Visual field tests conducted with the Octopus perimeter (Haag-Streit AG Köniz, Switzerland). The left panel shows the visual field of the left eye, displaying an upper nasal defect and a central scotoma. The right panel shows the visual field of the right eye, without significant defects.

Two years after SRT, the patient exhibited cotton wool spots along the inferior temporal vascular arcade, a fluorescein angiography was performed to investigate signs of perfusion disturbances. Consistent with the presence of exudates, a small block of background fluorescence was observed, together with mild pericapillary leakage ( Fig. 3 a ). The OCT-A (swept-source OCT-A Plex Elite 9000; Carl Zeiss Meditec, Oberkochen, Germany) analysis shows a wide area of non-perfusion with absence of vessels, both of superficial and deep plexuses, with extension towards the foveal zone ( Fig. 3 b – **c** ). Consistently, a consensual progressive reduction of the retinal nerve fiber layer (RNFL), ganglion cell layer (GCL), and IPL thickness was observed the second year post-treatment in the same retinal region. The internal retinal thinning initially involved the internal and external nasal EDTRS regions and subsequently, the lower EDTRS regions ( Fig. 4 ). The thickness of retinal layers was obtained using the automatic Spectralis segmentation module, with subsequent manual correction.

Four years following SRT, sectorial temporal atrophy of the left optic disc was observed for the first time. The sectorial disc pallor was associated with a physiological and symmetrical cup/disc ratio of 0.3, suggesting a non-glaucomatous etiology. Moreover, intraocular pressure was within normal limits at all visits the patient underwent at our center. Cerebral magnetic resonance imaging (MRI) was then performed to rule out other causes of optic neuropathy.

The morphological appearance of the optic disc remained unchanged up to the latest visit to our center, ten years after the SRT Treatment, with no progression of the optic atrophy or cupping. A papillary OCT confirmed a loss of peripapillary RNFL in the inferotemporal sector of the optic disc, and automated perimetry (G2 test strategy, Octopus, Haag-Streit AG, Köniz, Switzerland) showed defects in the upper nasal quadrant and a central scotoma ( Fig. 4 b ).

## Discussion

We presented a case of a radiation retinopathy associated with neuronal Wallerian anterograde degeneration with consequent sectorial optic disc atrophy. To the best of our knowledge, this is the only reported case of such clinical presentation following low-dose stereotactic radiation therapy for nAMD.

Radiation retinopathy is known to be a chronic and progressive condition affecting the retinal capillaries, primarily due to endothelial damage caused by ionizing radiation, which results in vascular incompetence and retinal ischemia. Clinical manifestations of radiation retinopathy often involve a variety of retinal changes, such as microaneurysms, macular edema, cotton wool spots, hard exudates, telangiectasia, and sheathing around the vessels 7 . Due to the precise targeting of radiotherapy with the IRay device (Oraya Therapeutics Inc., Newark, CA, USA) and the minimal retinal exposure area, the development of florid radiation retinopathy in patients treated with low-dose stereotactic radiation was not expected 6 . However, MVAs have been detected in up to one-third of patients in the INTREPID study after 3 years 6 , and a similar incidence was reported in another real-life study. It is plausible to assume that the incidence of MVAs in patients treated with low-dose stereotactic radiation is, so far, underestimated, as both studies did not include OCT-A as part of the investigation protocol. In fact, the significance of OCT-A in diagnosing early subclinical microvascular damage post-radiotherapy has been extensively demonstrated 8 .

In previous studies, MVAs following low-dose radiation for wet AMD have shown a predilection for the parafoveal inferior and inferior-nasal area of the macula 6 , 9 . It has been suggested that this localization may be attributed to the inferior entrance of all three radiation beams through the sclera, resulting in a higher radiation dose to the inferior part of the macula 9 . Consistently, our case exhibited the onset of cotton wool spots along the inferior temporal vascular arcade and OCT-A revealed a marked reduction in both deep and superficial capillary plexus density in the nasal and inferonasal macular region. In the same retinal areas, we observed a progressive reduction in the thickness of the RNFL, GCL, and INL starting from 2 1/2 years after radiation therapy. A spatial relationship between the localization of microvascular anomalies and a reduction in the thickness of the retinal layers in patients having undergone low-dose SRT was already observed in a retrospective study conducted at our department in 2013 involving 50 eyes 9 . The correlation between inner retinal layer thickness, retinal capillary density, received radiation dose, and visual function was recently investigated in a cohort of patients exposed to ocular brachytherapy, moving a step forward toward a more comprehensive understanding of the link between microvascular and neuronal damage resulting from radiation. Tamplin et al. propose that the primary damage occurs at the optic nerve head, leading to retrograde degeneration that results in thinning of the inner retina. Consequently, the observed reduction in capillary density of the inner retinal plexus is posited to be a reaction to the neuronal loss, following a mechanism of neurovascular coupling 10 . Conversely, in the clinical case presented, we hypothesize that the thinning of the inner retinal layers and, ultimately, the sectoral atrophy of the optic nerve are caused by anterograde degeneration following retinal ischemic insult due to radiation exposure. The post-laminar optic nerve is affected as a consequence of the radiation retinopathy 11 , presenting with a subtle, yet progressive, pallor of the optic nerve head. This anterograde axon degeneration is also called Wallerian degeneration 12 and it has been described in patients with glaucoma, retinal ischemia, retinal degeneration, or toxic processes.

Our hypothesis is substantiated by the lack of typical clinical manifestations suggestive of radiation optic neuropathy, such as optic disc edema, hemorrhages, acute vision loss, or, in milder instances, diffuse pallor of the optic nerve, observed during our patientʼs follow-up. Furthermore, the onset of microvascular anomalies precede the thinning of the inner retina by approximately 6 months. To better assess the temporal relationship between microcapillary damage and the reduction in the thickness of the inner retina, a longitudinal evaluation of the two retinal capillary plexuses using OCT-A before radiation treatment would have been decisive.

The patient presented with radiation-induced microvascular abnormalities 2 years after receiving low-dose SRT for nAMD. Five years later, OCT-A allowed us to obtain precise images of both the superficial and deep capillary plexuses and topographic correlate microvascular alterations with inner retinal thinning, sectoral disc atrophy, and functional defects. Prospective longitudinal studies are necessary to elucidate the intricate relationship between microvascular and neuronal retinal damage in patients undergoing radiation therapy.

In conclusion, this case highlights the importance of long-term multimodal imaging monitoring for patients who have undergone radiation therapy, as evidenced by the development of microvascular abnormalities and their impact on the retinal structure. Utilizing advanced imaging techniques such as OCT-A and tools such as segmentation with SD-OCT enables us to elucidate intricate anatomical-functional relationships, improving our diagnostic sensibility and patient management.
